# The body interior in anorexia nervosa: from interoception to conceptual representation of body interior

**DOI:** 10.3389/fpsyg.2024.1389463

**Published:** 2024-06-24

**Authors:** Aude Paquet, Murielle Girard, Céline Passerieux, Marie-Christine Boule, Aurélie Lacroix, Pierre Sazerat, Bertrand Olliac, Philippe Nubukpo

**Affiliations:** ^1^Department of Research and Innovation, Centre Hospitalier Esquirol, Limoges, France; ^2^INSERM, Univ. Limoges, IRD, U1094 EpiMACT, Institute of Epidemiology and Tropical Neurology, Limoges, France; ^3^University Paris-Saclay, UVSQ, Inserm U1018, CESP, Team DevPsy, Villejuif, France; ^4^University Hospital Department of Child and Adolescent Psychiatry, Centre Hospitalier Esquirol, Limoges, France; ^5^University Hospital Department of Addictology, Centre Hospitalier Esquirol, Limoges, France

**Keywords:** interoception, body interior representation, anorexia nervosa, body interior knowledge, drawing

## Abstract

**Background:**

Body image disorders are well documented in anorexia nervosa (AN); however, knowledge of interoceptive awareness (IA) in this population remains poor. This descriptive study investigated whether and how the representation of the interior of the body may have an impact on IA.

**Methods:**

The representations and knowledge of the body interior were evaluated with a drawing task in 34 women with AN and 34 healthy controls (HCs). A lexicometric analysis was performed on the vocabulary used to describe the drawn body parts in a structured interview. It was assumed that the conceptual representation of the body interior could be affected by or influence IA. Thus, the relationship between IA, measured with the heartbeat task and the ischemia-induction test, and the drawings was explored. Other scales, such as those of body shape, awareness or satisfaction, were used to assess affective representations of the body.

**Results:**

The drawing, lexicometric and IA results were similar in the two groups. No correlations were found among IA, body representation scores and representation level of body interior. Only the representation of bones by the AN group was significantly different.

**Discussion:**

Increased visual attention to the skeleton or greater awareness of bone health could explain the stronger representation of bones in the AN group. The psychophysical therapy received by some AN participants (73%) did not seem to have influenced IA. Our results do not support a relationship between IA and the representation of the body interior.

**Clinical trial registration:**https://clinicaltrials.gov/, identifier NCT03988218.

## 1 Background

Anorexia nervosa (AN) is a severe and persistent mental disorder associated with physical and psychosocial impairments (Zipfel et al., 2015). Distorted body representation (for a review, see Gadsby, 2017) is an important feature of AN and serve as both a symptom and a risk factor (Glashouwer et al., 2019).

Awareness of the body relies on strong multisensory integration. However, in anorexia, the perception of the body can deviate from sensory input, leading to dysmorphophobia. Pitron and de Vignemont (2017) suggested that internal representations of the body organize complex sensory information about the body. In this mental body model, the objective is to reliably and systematically translate the biological reality of the body by processing sensory, motor, emotional, and social information. The construction of the internal model occurs gradually by increasingly detaching from sensory inputs until it becomes disconnected (phantom limb experience) (Pitron and de Vignemont, 2017) and may be the origin of distorted body representations such as overestimation of body size significantly amplified in AN (Keizer et al., 2012).

Bodily perceptions include three sensory dimensions: proprioception (detecting signals from the musculoskeletal system), exteroception (detecting external stimuli through the five senses), and interoception (which involves the nervous system sensing, interpreting, and integrating signals originating from within the body) (Farb et al., 2015; Khalsa et al., 2018; Quadt et al., 2018; Khalsa et al., 2022). Several studies have demonstrated disruptions in the integration of somesthetic signals, especially tactile input (Keizer et al., 2011), and input from other bodily perception modalities [e.g., tactile-kinesthetic/haptic (Grunwald et al., 2001); pain (Papezová et al., 2005)]. Different dimensions of interoception, such as interoceptive accuracy, sensibility, attention, and awareness (Garfinkel et al., 2015) may have distinct effects on body awareness (Crucianelli et al., 2018).

Garfinkel and Critchley (2013) proposed a three dimensional construct of interoception that distinguishes interoceptive accuracy that refer to performance on objective behavioral tests, interoceptive sensibility that refer to assessment of subjective interoception, and interoceptive awareness that refer to metacognitive awareness of interoceptive accuracy. Dysfunctional interoception across various dimensions has been associated with AN (Jenkinson et al., 2018; Martin et al., 2019). Regardless of the assessment method, whether subjective (e.g., questionnaires) or objective (e.g., heartbeat perception tasks), individuals with acute AN tend to exhibit lower self-reported interoceptive awareness (IA) (Weineck et al., 2021; Phillipou et al., 2022) and reduced ability to accurately perceive bodily signals (Pollatos et al., 2008) and discriminate between hunger and satiety sensations (Fassino et al., 2004; Matsumoto et al., 2006). Impaired interoception, regardless of signal origin (gastric interoception, cardiac interoception or pain), has been observed in AN (Martin et al., 2019). Furthermore, impaired neural processing of interoception has been linked with eating disorders (Strigo et al., 2013).

Interoception has been re-conceptualized as a bidirectional process, influenced by both the brain and the body (Berntson and Khalsa, 2021). Originally, interoception was thought to be a one-way pathway, with ascending signals traveling from the body to the brain and descending regulatory responses. In this bidirectional model, feedback and feedforward loops continuously update the internal model to predict and regulate future states of the body (Petzschner et al., 2021). Sensory signals are thus constantly shaped and modified by the individual’s expectations. As interoception is a process that links the body and the brain, it is conceivable that evaluating this process could enhance understanding of cognitive or psychological functions (Herbert and Pollatos, 2012). From this bidirectional perspective, the conceptual representation (knowledge and semantics) of the internal anatomy of the body could be influenced by or influence interoceptive perception. Research on bodily signals and psychological processes supporting interoception needs to be conducted, and the relationship between interoception and conceptual representation of the interior of the body is not clear. The interior of the body representations has been assessed with drawings in individuals with chronic or somatic conditions (Tait and Ascher, 1955; Vessey and O’Sullivan, 2000; Catteau, 2010; Girard et al., 2011, 2015), but has never been assessed in eating disorders.

From the perspective that the brain and the body cannot be fully understood when studied separately, we conducted a descriptive study focusing on conceptual (body semantics), interoceptive (perception) and affective (body satisfaction) aspects of body perceptions.

This descriptive study investigated whether and how the representation of the interior of the body may have an impact on interoception. We used experimental tests to detect internal bodily sensations (the heartbeat tracking task; an induction of ischemia test) and to assess representations of the interior of the body (the Inside-of-the-Body Drawing Task). We used self-report questionnaires to assess interoceptive sensibility and affective dimensions of the body.

Given some of the sensory disturbances and poor body image described in anorexia, we hypothesized that interoception would be lower in participants with anorexia and expect them to score lower on drawings of the interior of the body.

## 2 Materials and methods

### 2.1 Aims and design of the study

Our aim was to describe the knowledge of the interior of the body using an adapted version of the Inside-of-the-Body Test (Tait and Ascher, 1955), a drawing task, in 34 women with AN and 34 age-matched healthy controls (HCs). We first compared their results. We also measured interoception from objective and subjective measures. Interoceptive accuracy and sensibility with the heartbeat tracking task, the ischemia induction test (Girard et al., 2011, 2015) and describe results in the AN and HC.

As a secondary aim, we investigated the relationships between interoception and drawing score in each group.

### 2.2 Participants

Participants were inpatients with AN hospitalized in the Esquirol Hospital Center in Limoges, France with a body mass index above 14 kg/m^2^. They were diagnosed by their referent psychiatrist according to the Diagnostic and Statistical Manual for Mental Disorders, 5th edition (DSM-5). Twenty-six participants have restrictive AN, seven have purgative AN, and one participant lacked information regarding the type of AN. HCs were recruited with posters placed in the Faculty of Humanities of Limoges (France) and by word of mouth in the Esquirol Hospital’s administrative staff rooms. The inclusion criteria for HCs were as follows: no psychiatric history, current organic/neurologic disease, or eating disorders and a body mass index between 18.5 and 24.9 kg/m^2^ (i.e., normal weight).

In both groups (AN and HC), participants were women, aged 14 years or older, without upper limb injury or pathology and were able to answer questionnaires and understand French; moreover, they were not pregnant or recruited from populations whose authority or rights are under legal protection. Participants in the two groups were matched in terms of age and level of education. Informed written consent was obtained from all participants or their parents, agreement was obtained from those less than 18 years old, and the study was approved by a French Committee for the Protection of the Persons, as required by French regulations (Clinical Trials number NCT03988218, registration 2019-03-25). The study was carried out in accordance with the principles of the Declaration of Helsinki.

### 2.3 Clinical and psychometric evaluations

#### 2.3.1 Body mass index (BMI)

BMI was used to estimate the actual body shape of participants.

#### 2.3.2 Eating attitude test (EAT-26)

The EAT is a questionnaire extensively used to assess disordered food attitudes (Garner and Garfinkel, 1979). It measures eating-related preoccupations and behaviors. The EAT includes 26 items with six response options, from infrequently/almost never/never (0) to always (Glashouwer et al., 2019), with a range of 0–78 points. A score of 20 or higher indicates possible cases of disturbed eating attitudes.

### 2.4 Conceptual representation of the body interior

#### 2.4.1 Inside-of-the-body drawing task

Assessment of body image distortion often involves subjective methods such as questionnaires or body drawings, which can be considered a form of self-report of bodily experiences (Kalisvaart et al., 2018). However, there is no validated questionnaire or scale for representations of the body interior.

In one experimental task, based on Tait and Ascher (1955) body drawing task, participants were asked to draw the interior of the body. The procedure was standardized in this study. Participants were asked to draw the interior of the body, including all body parts, and then label the body parts drawn. Participants were informed that they had a maximum of 5 min to complete the drawing. The drawing was made on an A4 sheet of paper presented vertically on which the outline of a person was drawn (Appendix 1). After the drawing, participants were asked to express their knowledge of the body parts they had drawn. For each body part drawn the following question was asked “Do you know what the (part drawn) is used for, or what function it is associated with?” Answers were transcribed verbatim.

A detailed rating scale was developed to guide the subjective analysis of the drawings. Details of the rating scale are provided in the Supplementary File. The rating scale included both qualitative and quantitative measures. The drawing score took into account the detail and accuracy of the body parts drawn and their location (Appendix 2). From a qualitative perspective, drawings were scored in terms of the system to which a body part belonged as well as the organization of this system (dispersed elements, organization). Drawings were scored blindly by two trained raters, both psychomotor therapists (scoring detailed in Supplementary File).

#### 2.4.2 Lexicometric analysis of the interviews

After each drawing, participants were interviewed about their functional knowledge of each body part drawn. The interviews were organized around the body parts drawn by the participant. All the responses were transcribed verbatim and formatted to carry out a lexicometric analysis, which was based on the method defined by Reinert (1983, 2003) and conducted using IRAMUTEQ^®^. This method, based on a descending hierarchical classification, allows evaluation of the organization and content of a representation (Abric, 2003). It reveals the lexical worlds that structure a text. The underlying hypothesis is that the words and terms used indicate an underlying representation and reveal relationships. During the analysis, statements are examined in terms of distribution/classification according to the redundancy of terms with a chi-square test. The interviews were coded according to the criteria used to distinguish the population interviewed (AN, HC) and the type of body part represented (e.g., brain and heart).

### 2.5 Interoceptive accuracy and interoceptive sensibility assessment

#### 2.5.1 Heartbeat tracking task

The heartbeat tracking task (HBTT) was used to evaluate interoceptive accuracy of an individual to her cardiac activity, which is part of interoceptive accuracy (Barrett et al., 2004; Critchley et al., 2004). This method is frequently used in individuals with eating disorders (Pollatos et al., 2008; Herbert and Pollatos, 2014). The level of individual interoceptive accuracy can be conceptualized as a trait-like sensitivity to visceral signals. The HBTT used here was a modified version of the Mental Tracking Method (Schandry, 1981) (see below).

Heart rate was recorded with a Polar heart rate monitor (model RS 800CX) (Ricciardi et al., 2016; Crucianelli et al., 2018), with a chest strap. The chest strap was connected to a monitor controlled by the experimenter. The data were then transferred to a computer via Polar Pro Trainer software.

Participants were instructed to sit comfortably, with their forearms and hands resting on their thighs, and relax without talking for at least 30 s before the task started. During the task, they were instructed to count their heartbeats silently. They were asked to concentrate on only their heartbeats and were not permitted to take their pulse or to attempt any other physical manipulations, which could facilitate detection.

There were four counting periods of 15, 25, 35, and 45 s in length, randomly assigned among participants of each group. The ‘start’ and ‘stop’ signals for each counting period were provided by the experimenter. After the stop signal, participants verbally reported the number of counted heartbeats. Participants were not informed about the length of the counting periods or their performance. IA was estimated as the mean heartbeat perception score according to the following equation:

1/4 ∑ [(1− (|recorded heartbeats – counted heartbeats|/recorded heartbeats))].

#### 2.5.2 Ischemia-induction task

The other test used to assess interoception was the induction of ischemia. The experimental procedures used are described in detail in a previous study on the induction of moderate pain (Girard et al., 2011, 2015; Paquet et al., 2019). A blood pressure cuff was inflated to a pressure greater than 10% of the peak systolic pressure, and the participant was asked to evaluate their arm sensation every minute using a visual analog scale (VAS). The test was stopped when the score reached 3 and the blood pressure cuff was deflated. The time taken to report a pain score of 3 on the VAS was recorded. The maximum time allowed for the test was 960 s.

#### 2.5.3 Immediate interoceptive perception task

Immediate interoceptive perception was evaluated with a custom-designed self-report questionnaire concerning general sensations of the internal body related to specific organs (heart, muscles, and viscera) or related to body perceptions of interoceptive origin such as breathing, muscle tone, and pain. The sensations were classified into seven categories: general, muscular, tonic, respiratory, pain, cardiac, and abdominal. Participants were asked about the sensations (one or more) they immediately experienced for each of these categories. The response options varied from “absence of bodily sensations” to several adjectives suggesting “presence of bodily sensations” (Appendix 3).

### 2.6 Affective representations of the body

#### 2.6.1 Body shape questionnaire (BSQ)

The BSQ was used to identify various aspects of dissatisfaction or concern with weight and body image in the 4 weeks prior to the interview; this self-report questionnaire evaluates body image and body dissatisfaction. The BSQ includes 34 items rated on a Likert-type scale ranging from 1 (never) to 6 (always). Higher scores denote greater levels of body dissatisfaction (Cooper et al., 1987; Rousseau et al., 2005).

#### 2.6.2 Body awareness questionnaire (BAQ)

The BAQ is a self-report questionnaire used to measure bodily sensitivity. It assesses attentiveness to normal, non-emotive internal bodily processes and sensations, specifically sensitivity to bodily cycles and rhythms, small changes in normal functioning, and anticipation of bodily reactions (Shields et al., 1989). It contains 18 items that are rated on a 7-point Likert scale ranging from 1 (not at all true about me) to 7 (very true about me).

#### 2.6.3 Body satisfaction and global self-perception questionnaire (QSCPGS)

The “Questionnaire de Satisfaction Corporelle et de Perception Globale de Soi” [Body Satisfaction and Global Self-Perception Questionnaire] (QSCPGS) is a French questionnaire composed of two series of 10 items evaluating how participants perceive their body and feelings regarding to global perceptions of self. Each item is composed of a positive term (e.g., calm) and an opposite term (nervous) on either side of a continuum centered at 0 and ranging from −1 to −5 and 1 to 5 on the left and right sides, respectively. In this scale, |1| corresponds to “very little,” and |5| corresponds to “very strong.” The total score ranges from −100 to +100, with each of the 20 items ranging from −5 to +5.

### 2.7 Procedure

Patients with AN and HCs were individually assessed by a therapist trained on the tests. The Inside-of-the-Body Drawing Test and the questionnaires were carried out in the same order (collection of sociodemographic and clinical data, BMI, EAT-26, HADS, Inside-of-the-Body Drawing Test, BSQ, QSCPGS and BAQ) to prevent the drawings from being influenced by questionnaire content. The HBTT and the ischemia- induction tasks were administered in a random order after the drawing test and questionnaires.

### 2.8 Statistical analysis

Statistical analyses were conducted using Statistical Package for Social Science (SPSS^®^) version 27 for Windows. Categorical variables are presented as percentages; quantitative variables are presented as the mean and standard deviation. Non-parametric analyses were used because of the non-normal distribution of data, as confirmed with the Shapiro–Wilk test. Comparisons of categorical variables were performed using the Pearson chi-square test, and comparisons of the means of quantitative variables between groups were performed using the non-parametric Mann–Whitney U test. Correlations between clinical variables and interoceptive scores or drawing scores were evaluated using Spearman’s correlation analyses. Verbatim reports on body knowledge were analyzed with a qualitative analysis using Iramuteq version 0.7 alpha 2 (software for textual data analysis or textual statistics on text corpora) (Ratinaud, 2009).

## 3 Results

### 3.1 Description of the population

Sociodemographic characteristics are presented in Table 1. Both groups were similar in age and sex by design. The clinical characteristics are reported in Table 2. BMI, treatments and EAT scores differed between the two groups. AN participants received more psychophysical care.

**Table 1:** Sociodemographic characteristics of the participants according to group.

	AN (*n* = 34)	HC (*n* = 34)	*p-*value
Age, years (mean ± SD)	24.38 ± 7.30	24.47 ± 7.33	Z = −0.55, *p* = 0.96
Level of education			
Secondary school, in progress	7	7	*Χ*^2^ = 0.18, dl = 3, *p* = 0.98
Vocational degree	5	6	
Associate’s degree	6	5	
University degree	16	16	
Professional category			
Student	17	17	*Χ*^2^ = 0.36, dl = 4, 0.36
Blue-collar worker	11	10	
Service worker	2	0	
White-collar worker	2	6	
Other	2	1	

**Table 2:** Clinical characteristics of the participants according to group.

	AN (*n* = 34)	HC (*n* = 34)	*p-*value
BMI, kg/m^2^	15.72 ± 1.25	24.68 ± 1.83	**<0.0001**
Medical history (n)	14	11	0.61
Sexual abuse history (n)	8	2	0.08
Treatment (*n*)			
Antidepressant	14	0	**<0.0001**
Anxiolytic	7	0	**0.01**
Hypnotic	9	0	**<0.01**
Various (painkillers, vitamins, and food supplements)	24	7	**<0.0001**
Psychophysical care			
Psychomotor therapy	17	0	**<0.0001**
Relaxation	8	3	
Both: psychomotor therapy and relaxation	2	0	
EAT score (mean ± *SD*)	40.68 ± 14.74	3.21 ± 3.11	**<0.0001**

### 3.2 Affective representation of the body

Patients with AN were less satisfied with their body shape than HCs (BSQ and QSCPGS scores), and HCs had a better awareness of their body (BAQ scores) (Table 3).

**Table 3:** Body awareness and body satisfaction of participants according to group.

Scale (mean ± *SD*)	AN (*n* = 34)	HC (*n* = 34)	*p-*value
BSQ score	126 ± 36.53	58.29 ± 18.56	**<0.001**
BAQ score	79.41 ± 23.95	93.71 ± 21.23	**0.015**
QSCPGS score	−12.68 ± 34.26	49.74 ± 23.47	**<0.001**

### 3.3 Conceptual and interoceptive representation of the body: experimental tests

#### 3.3.1 Interoception tasks

The HBTT score and scores on ischemia-induction task did not differ between the AN and HC groups (Table 4). The immediate interoceptive perception score was higher in the AN group (*p* = 0.04), especially for muscular and abdominal perceptions (Z = −2.143, *p* = 0.032; Z = -5.532, *p* < 0.001). There was no correlation between clinical variables (age, BMI, BSQ score, BAQ score, or QSCPGS score) and interoceptive task scores for each groups. In all participants, there was no correlation between the interoceptive task scores and the drawing scores.

**Table 4:** Performance on the interior of the body representation test and interceptive perception tests of two participants according to group.

Task (mean ± *SD*)	AN (*n* = 34)	HC (*n* = 34)	*p-*value
Inside-of-the-Body Drawing Task			
Drawing total score	15.12 ± 8.19	15.96 ± 7.63	0.52
Number of body parts drawn	15.24 ± 8.21	12.91 ± 6.41	0.30
Immediate interoceptive perception score	5.5 ± 1.54	4.97 ± 1.36	**0.04**
Heartbeat tracking score	0.57 ± 0.26	0.53 ± 0.27	0.51
Ischemia-induction task			
Maximum VAS score	4.1 ± 1.62	3.99 ± 1.54	0.19
Maximum duration	2.94 ± 3.55	3.97 ± 4.46	0.35

Concerning drawing task, there was no difference between the two groups (total score or number of body part drawn, see Supplementary File) (Table 4).

There was no correlation between clinical variables (age, BMI, BSQ score, BAQ score, or QSCPGS score) and drawing total score or number of body parts drawn for each groups. Only the level of education was positively correlated with the drawing total score (*r* = 0.33; *p* = 0.006).

There was no difference in drawing score between participants with and without a medical history. However, participants with AN receiving psychophysical care drew significantly more body parts than participants without this care (*p* = 0.01).

Regarding the representation of body parts, patients with AN drew less distorted or better organized bone drawings than HCs (*p* = 0.04). Analysis according to psychophysical care status or BMI score in all participants (patients with AN + HCs) showed differences in bone representation. Participants with low BMI had higher scores in bone representation among all participants (*r* = −0.255, *p* = 0.036). Participants receiving psychophysical care had higher bone drawing scores (*p* = 0.005); however, no such pattern was found among patients with AN (*p* = 0.25).

Analysis of drawings according to physiological system (e.g., central nervous, cardiovascular, respiratory, or gastrointestinal system) did not reveal any differences between the two groups (Appendix 4). However, the musculoskeletal system tended to be better organized in participants with AN (*U* = 424.5; *p* = 0.053).

#### 3.3.2 Knowledge of the interior of the body

**Figure 1:**
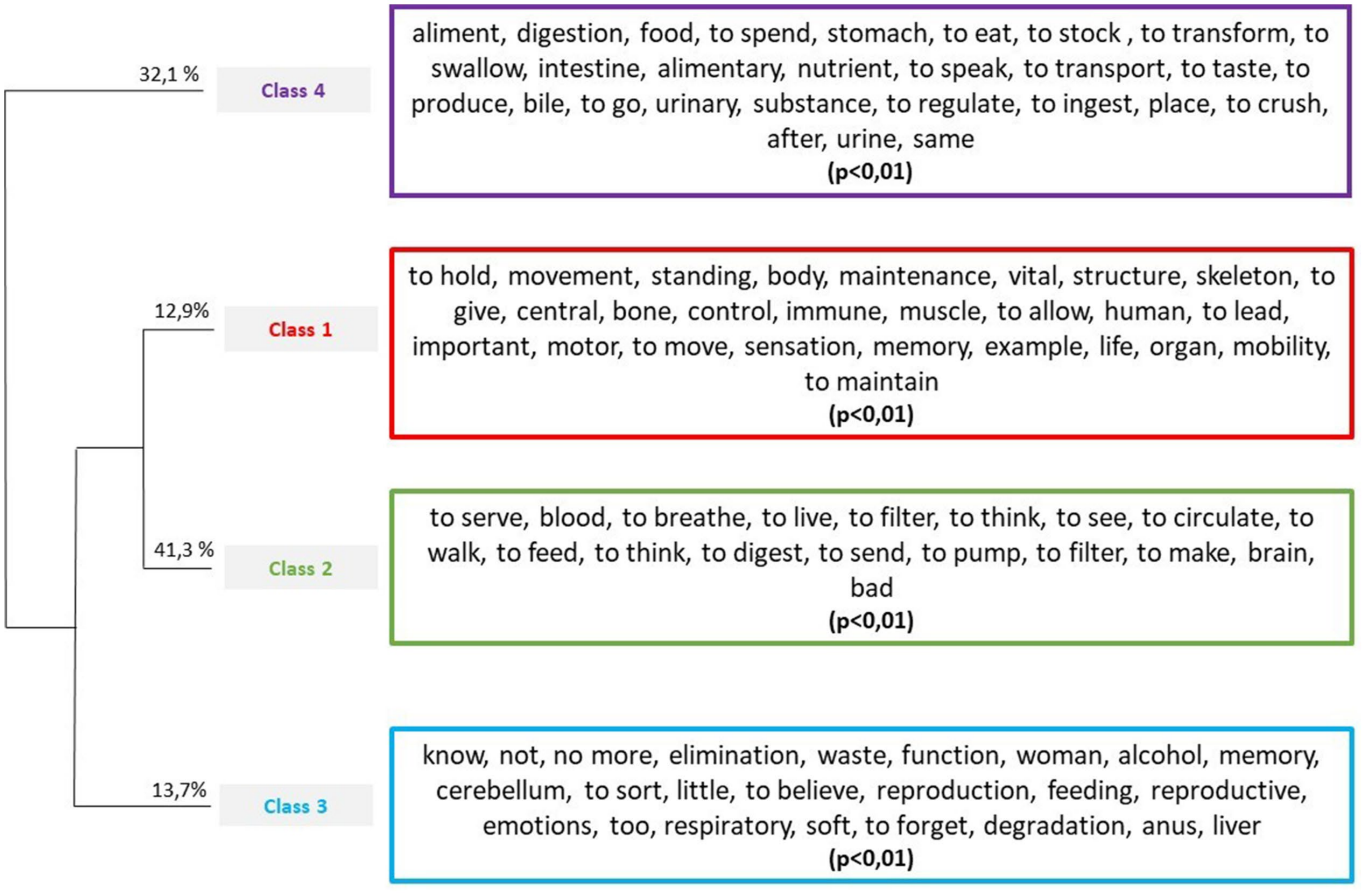
Hierarchic ascending classification with significant lexical forms (*p* < 0.01).

Figure 1 shows a graphical representation of the distribution of lexical clusters in the text corpus across all body parts and participants. First, we note that class 4 differs from the other classes. It represents 32.1% of the classified words. This class centers on food and digestion. The variables stomach (*χ*^2^ = 68.46; *p* < 0.0001), small intestine (*χ*^2^ = 16.18; *p* < 0.0001), tongue (*χ*^2^ = 14.74; *p* < 0.001), intestines (*χ*^2^ = 11.79; *p* < 0.001), teeth (*χ*^2^ = 10.66; *p* < 0.001), trachea (*χ*^2^ = 8. 4; *p* = 0.003), colon (*χ*^2^ = 7.3; *p* = 0.006), gastrointestinal tract (*χ*^2^ = 4.24; *p* = 0.04), throat (*χ*^2^ = 4.24; *p* = 0.04), pancreas (*χ*^2^ = 3.86; *p* = 0.05), and large intestine (*χ*^2^ = 3.86; *p* = 0.05) were associated with class 4.

Classes 1 and 2 consist of cardiorespiratory function and brain function (class 2) as well as maintenance and movement associated with the structure of the body (class 1). The following variables were associated with class 1: bones (*χ*^2^ = 140.25; *p* < 0.0001), muscles (*χ*^2^ = 17.35; *p* < 0.0001), brain (*χ*^2^ = 8.3; *p* = 0. 004), spine (*χ*^2^ = 7.75; *p* = 0.005), ribs (*χ*^2^ = 6.76; *p* = 0.009), AN (*χ*^2^ = 6.16; *p* = 0.013), and spleen (*χ*^2^ = 4.93; *p* = 0.026). The following variables were associated with class 2: lungs (*χ*^2^ = 62.58; *p* < 0.0001), heart (*χ*^2^ = 53.08; *p* < 0.0001), eyes (*χ*^2^ = 10.07; *p* = 0.001), brain (*χ*^2^ = 9.62; *p* = 0.002), veins (*χ*^2^ = 7.17; *p* = 0.007), and eye (*χ*^2^ = 4.29; *p* = 0.04).

Finally, class 3 consists of vocabulary related to the absence of knowledge (e.g., “I do not know”) as well as elimination and reproduction. The following variables were associated with class 3: liver (*χ*^2^ = 28.35; *p* < 0.0001), uterus (*χ*^2^ = 20.64; *p* < 0.0001), pancreas (*χ*^2^ = 8.08; *p* = 0.004), ovaries (*χ*^2^ = 6.74; *p* = 0.009), gallbladder (*χ*^2^ = 6.3; *p* = 0.012), and cerebellum (*χ*^2^ = 3.98; *p* = 0.046).

**Figure 2:**
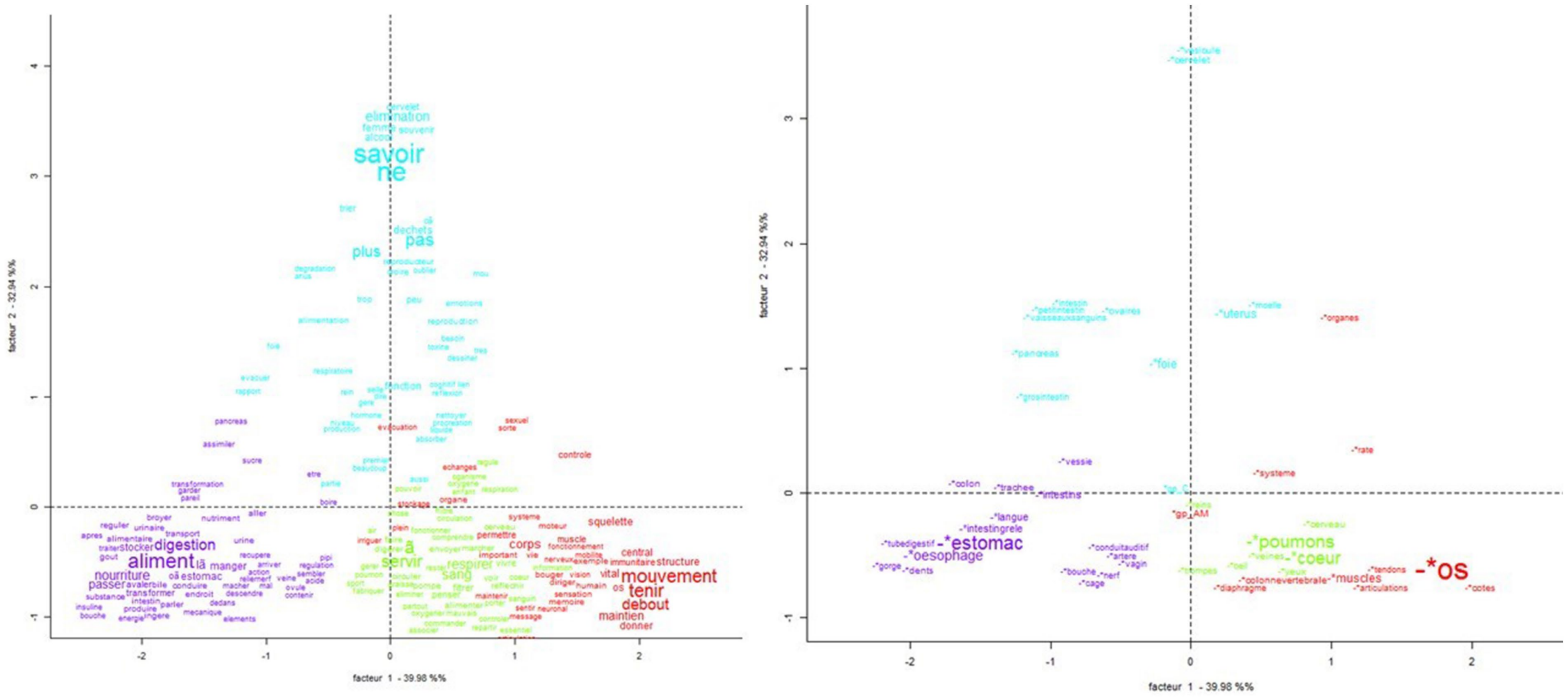
Correspondence analysis plot of the most frequent active French words in each of the lexical classes obtained in the descending hierarchical classification. Sizes of active words are proportional to their frequency in the text. Colors correspond to the lexical classes (Class 1: red; Class 2: green; Class 3: blue; Class 4: purple).

The factorial correspondence analysis (CA) (Figure 2) shows the level of knowledge along the *y*-axis, with “do not know” as positive and knowledge associated with body parts as negative. Food is shown on the x-axis, with body parts and vocabulary not related to food (e.g., “bone,” “movement,” and “hold”) as positive and body parts or vocabulary related to digestion and food (e.g., “aliment,” “food,” “digestion,” and “stomach”) as negative. The AN variable was significantly associated with class 1, and the HC variable was significantly associated with class 3. These two variables are clustered in the center of the CA around the two axes, suggesting no opposing characteristics.

## 4 Discussion

In this study, we focused on representations of the body interior in AN from conceptual, affective, and perceptive approaches. We used non-verbal assessments to evaluate interoception and body knowledge.

We assessed representations of the body interior and body knowledge with a modified version of the Inside-of-the-Body Drawing Test. However, comparison of the drawings did not identify any differences between the AN and HC groups. Drawing total scores and the numbers of internal body parts drawn were similar between the two groups, suggesting a comparable representation of the body interior. Participants with AN and HCs had similar anatomical knowledge, mostly drawing cardiac (95%) and visceral organs (93% drew lungs and 79% drew the stomach). The level of representation (organization, detail level) of each body part differed only in bones between the groups. Participants with AN drew bones in greater detail (more precise and better organized or localized drawings), suggesting a higher level of representation of bones in AN. Analysis according to medical history (fractures, head injuries, and sprains) did not explain this difference between the two groups. Several hypotheses may explain the higher representation of bones among participants with AN. For example, increased visual attention to the skeleton due to low weight or cachexia seems possible because of the identified links between a low BMI and higher bone representation scores. In addition, a greater awareness of bone health because of the considerable negative impact of AN on the growing skeleton (Mumford et al., 2019) could also explain this stronger representation. Indeed, participants with AN are very conscious of the physiological consequences of undernutrition and took various steps to address this issue, such as vitamins and dietary supplements.

With the exception of a more elaborate representation of bones in the AN group, people with AN had subjective representations of the body interior similar to people without an eating disorder of the same age, sex, and education level. While AN patients exhibit body image distortion, representations of the body interior were not altered in our study. Body image is commonly assessed with self-report of bodily dimensions by body size estimation tasks (Mölbert et al., 2017). The underlying assumption of these tasks is that comparative size judgments are obtained through a comparison process: perceptual content (derived from the stimulus) is compared to relevant content stored in the body image (Gadsby, 2019; Brown et al., 2021). Based on this hypothesis, we studied interoceptive perception using a heartbeat perception task. Similar to Richard et al. (2019), we found that the AN and HC groups exhibited similar heartbeat perception. Although use of the heartbeat perception task has been debated (Eshkevari et al., 2014; Ring and Brener, 2018) and does not represent interoceptive awareness as a whole, it is still widely used as an objective indicator of interoceptive accuracy. Specifically, Pollatos et al. (2008) reported weaker heartbeat perception in the AN group, while Khalsa et al. (2015) reported a higher likelihood of detecting interoceptive sensations in the AN group. In our condition, we did not found link between interoception experimental tasks and interoceptive sensibility evaluated by self-report questionnaire. In a non-clinical sample of young adult, Garfinkel et al. (2015) observed relationships between interoceptive accuracy and subjective interoceptive sensibility only in people with high interoceptive accuracy. These heterogeneous results regarding the assessment of interoception in AN population suggest that further studies are needed. In the ischemia-induction task, the AN and HC groups exhibited comparable ability to detect changes in a weakly innervated organ. The psychophysical therapy received by some AN participants did not influence their ability to detect interoceptive signals. Moreover, perceptive judgments and representations of the body interior were adequate in both groups, without specific differences in the AN group. Contrary to our prediction, we did not find evidence that interoceptive accuracy or interoceptive sensibility was related to representations of the body interior. To our knowledge, no other study has previously investigated whether interoception is related to representations of the body interior.

Studies have indicated that body image is influenced by cognition (beliefs about body shape or appearance and the mental representation of one’s own body), perception and affect (feelings of satisfaction) (Gadsby, 2017; Brown et al., 2021). Regarding this perspective, we did not find a relationship between drawing scores and body shape, body awareness or body satisfaction. Although the representation of one’s own body, body awareness and body shape differed between the AN and HC groups, these differences did not seem to affect the representation of the body interior.

However, detailed analysis of the vocabulary associated with the function of each body part drawn yielded interesting results. We found no studies on anatomical and functional knowledge associated with representations of the body interior in eating disorders. The lexicometric analysis of the function of each body part allowed us to evaluate knowledge of internal organs and body parts. The results, which did not differ between the two groups, showed that vocabulary associated with “food” and “digestion” was associated with organs of the digestive system (“stomach,” “esophagus,” and “intestine”), suggesting that the representations of the body interior are mainly visceral representations related to the functions of feeding. The factorial correspondence analysis (CA) confirmed a more pronounced representation of bone elements in participants with AN, associated with words focused on maintenance and movement associated with “bones,” “muscles,” and “brain” and the musculoskeletal system. The CA revealed no differences in characteristics between the AN and HC groups, suggesting that their knowledge of the body parts drawn was comparable.

### 4.1 Limitations

This study has several limitations that warrant consideration. First, this study had a small sample size; the sample consisted solely of women, and the study did not provide information on men’s characteristics. Although the drawing task was standardized and the results were compared with a control group, the task was experimental and not a validated psychometric instrument. The detailed description of the testing and scoring procedure can, however, be used to support future studies. In this task there is a person already outlined on the paper and this silhouette pertains to an ideal normal-weight person. The body distortion present in individuals with AN could interfere with filling in the silhouette. Moreover, other types of tests based on several organ systems (e.g., gastrointestinal, urinary, and respiratory intensity) could be more informative in future studies, as they specifically assess organ-specific concepts and allow generalization of IA. The conflicting findings regarding BAQ scores and IA in our experimental tasks as well as inconsistencies among studies highlight the need to explore the interaction between interoception and body image disruption in AN.

Indeed, despite theoretical advances and evidence supporting the idea that the brain and the body cannot be fully understood when studied separately, most explanatory neuroscientific approaches attempting to understand cognitive, emotional, and behavioral functioning in patients with eating disorders have assessed them separately.

## 5 Conclusion

To our knowledge, this exploratory study is the first to examine a link between interoceptive perception and representations of the body interior in patients with eating disorders. Similarities were found between women with and without AN concerning interoceptive perception and representations of the body interior. No link was found between interoception and representations of body interior in either the AN or HC groups. Analysis of drawings of the interior of the body revealed an overrepresentation of skeletal elements in AN participants; these skeletal elements were associated with maintenance, movement, and support of the body. These results provide a new avenue for exploring interoceptive perception in people with AN and suggest a standardized drawing task that may be used to explore this interoceptive experience.

Body drawings, as a form of self-report of bodily experience, may be appropriate because drawings rely less on conscious reflective mechanisms of the brain, are non-intrusive and are quick and easy to administer (Betts, 2006). However, the utility of drawing a person or a figure of the self, as a projective assessment technique, has been debated. Indeed, the scientific evidence for the validity and reliability of drawings as a reflection of psychological characteristics is weak (Betts, 2006). However, when body image and physiological issues are assessed, drawings have been found to be of value (Clarke and Newell, 1997; Guez et al., 2010; Kalisvaart et al., 2022). For example, Guez et al. (2010) showed differences between people with and without eating disorders using a self-figure drawing test. However, their assessment was not comparable with our tool because the drawing test used did not focus on the interior of the body. We did not find studies about representations or knowledge of the interior of the body that used a drawing task with patients with eating disorders. Although the drawing task used in the present study was not a validated psychometric assessment characterizing a psychological dimension of body image, it allowed us to evaluate individual representations of the body interior of women with AN.

## Data availability statement

The raw data supporting the conclusions of this article will be made available by the authors, without undue reservation.

## Ethics statement

The studies involving humans were approved by Comité de Protection des Personnes, Ouest V. The studies were conducted in accordance with the local legislation and institutional requirements. Written informed consent for participation in this study was provided by the participants’ legal guardians/next of kin.

## Author contributions

AP: Conceptualization, Formal analysis, Funding acquisition, Investigation, Methodology, Project administration, Software, Validation, Writing – original draft, Writing – review & editing. MG: Methodology, Supervision, Writing – review & editing. CP: Investigation, Writing – review & editing. M-CB: Investigation, Writing – review & editing. AL: Resources, Writing – review & editing. PS: Investigation, Resources, Writing – review & editing. BO: Supervision, Writing – review & editing. PN: Resources, Supervision, Writing – review & editing.
