# Supplementary material for: The body interior in anorexia nervosa: from interoception to conceptual representation of body interior
Source: Front Psychol. 2024 Jun 24;15:1389463. doi: 10.3389/fpsyg.2024.1389463 (PMC11229774; doi:10.3389/fpsyg.2024.1389463)
Supplement: Supplementary file 1 [file Data_Sheet_1.PDF]

Appendix

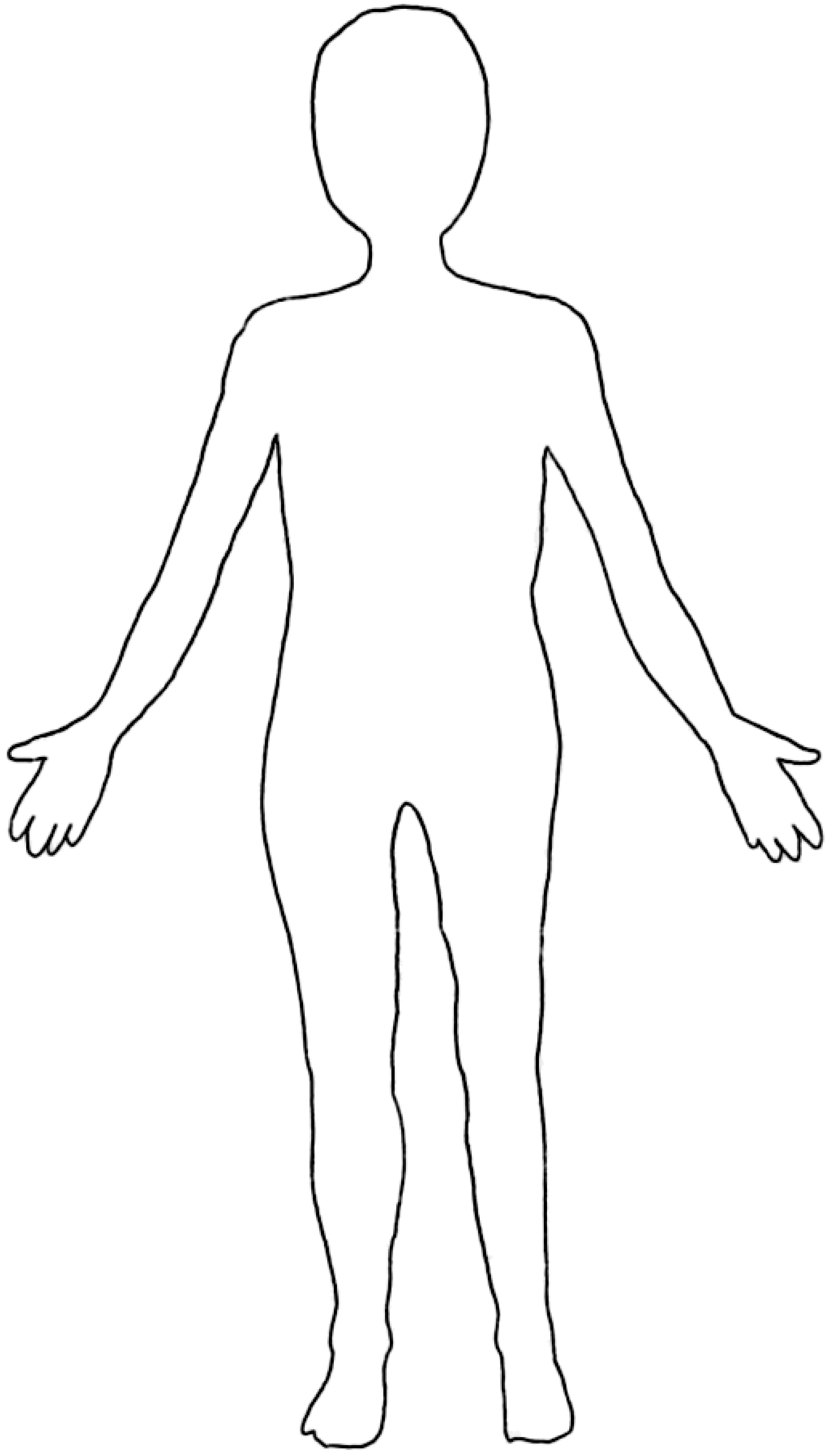

A1: Outline of a person used in the Inside-of-the-Body drawing task

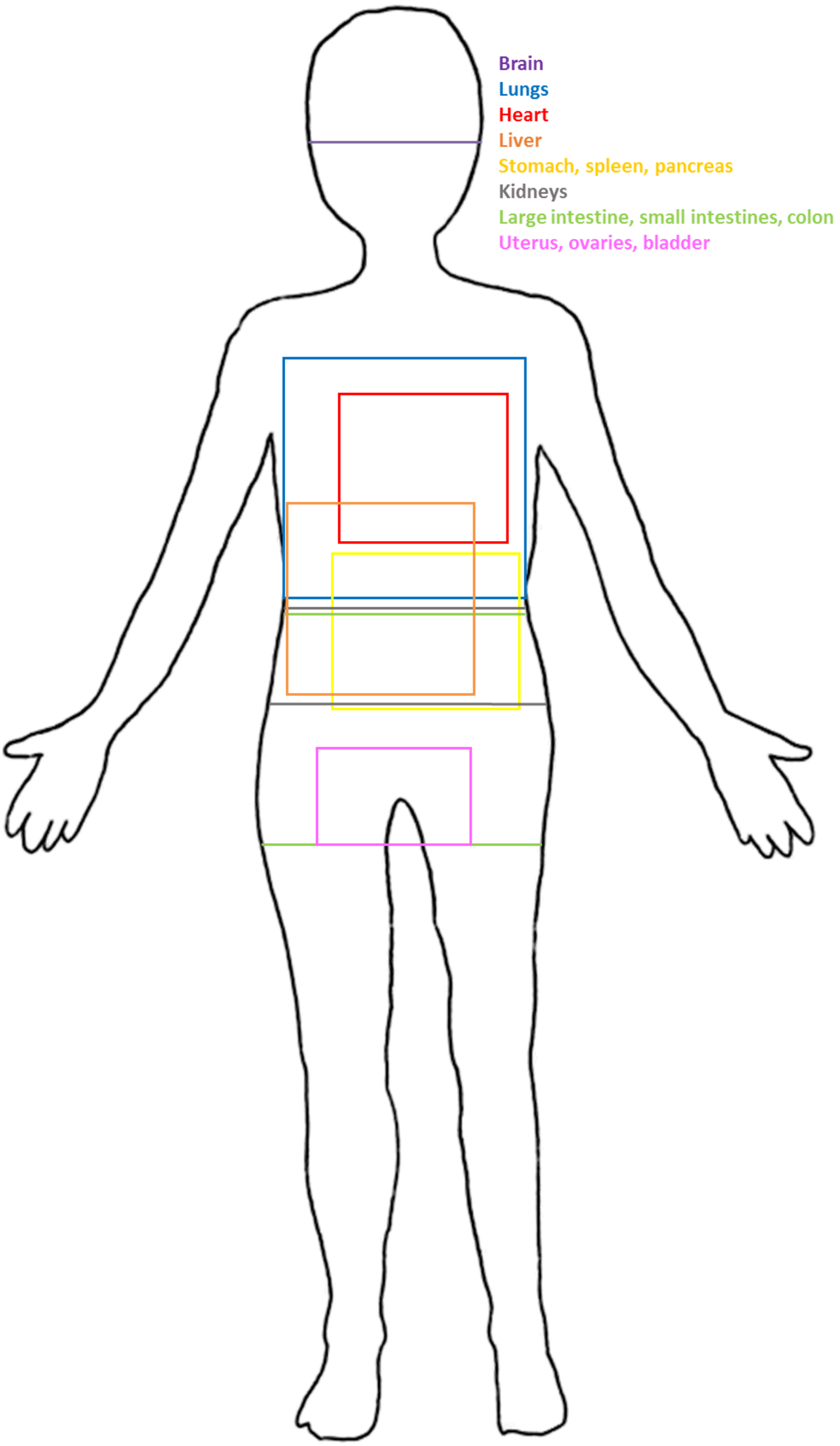

A2: Outline of a person and the regions of organs used to evaluate the localization of organs for scoring transparency

This questionnaire is designed to allow you to express what you perceive in terms of internal (bodily) sensations.

Before answering the questions, we ask that you pay attention to the bodily sensations you may be feeling right now.

	Specific sensations				
**In general, how do you feel at the moment?**	☐ Secure	☐ Uneasy	☐ Comfortabl e	☐ Uncomfortab le	☐ No specific sensation
**At the muscular/skeletal level, how do you feel?**	☐ Contracted<br>☐ Cramped<br>☐ Flexible	☐ Released<br>☐ Numb<br>☐ Stiff	☐ Softened<br>☐ Active<br>☐ Creaky	☐ Relaxed<br>☐ Painful<br>☐ Achy	☐ No specific sensation
**On a tonic level, how do you feel?**	☐ Tense<br>☐ Light<br>☐ Tired	☐ Full of energy<br>☐ Febrile<br>☐ Weak	☐ Crisp<br>☐ Tonic<br>☐ Heavy	☐ Relaxed<br>☐ Numb<br>☐ Active	☐ No specific sensation
**On a respiratory level, how do you feel?**	☐ Suffocated<br>☐ Regular	☐ Fluent<br>☐ Oppressed	☐ Panting<br>☐ Asphyxiate d	☐ Calm<br>☐ Jerky	☐ No specific sensation
**In terms of pain, how do you feel?**	☐ Severe<br>☐ Chronic	☐ Sharp<br>☐ Twinge	☐ Tingling	☐ Intermittent	☐ No specific sensation
**At the abdominal/visceral level, how do you feel?**	☐ Gurgling<br>☐ Labored<br>☐ Stomach knots	☐ Comfortable<br>☐ Bloated<br>☐ Heavy	☐ Noisy<br>☐ Burning<br>☐ Tense	☐ Full<br>☐ Disgusted<br>☐ Cramped	☐ No specific sensation
**At the heart level, how do you feel?**	☐ Rapid	☐ Labored	☐ Regular		☐ No specific sensation

A3: Immediate interoceptive perception task

Body part	AN		HC		All	
	n	%	n	%	n	%
Heart	33	97.1	32	94.1	65	95.6
Lungs	31	91.2	32	94.1	63	92.6
Stomach	29	85.3	25	73.5	54	79.4
Brain	23	67.6	29	85.3	52	76.5
Liver	22	64.7	27	79.4	49	72.1
Intestines	19	55.9	20	58.8	39	57.4
Bones	22	64.7	14	41.2	36	52.9
Kidneys	11	32.4	15	44.1	26	38.2
Muscles	14	41.2	11	32.4	25	36.8
Esophagus	13	38.2	11	32.4	24	35.3
Uterus	9	26.5	12	35.3	21	30.9
Bladder	8	23.5	12	35.3	20	29.4
Large intestine	6	17.6	9	26.5	15	22.1
Small intestine	9	26.5	6	17.6	15	22.1
Trachea	7	20.6	6	17.6	13	19.1
Pancreas	6	17.6	7	20.6	13	19.1
Tongue	6	17.6	7	20.6	13	19.1
Arteries and veins	3	8.8	8	23.5	11	16.2
Ovaries	3	8.8	6	17.6	9	13.2
Spleen	4	11.8	5	14.7	9	13.2
Colon	5	14.7	3	8.8	8	11.8

A4 Proportion of most represented body parts by group
