# Supplementary material for: The body interior in anorexia nervosa: from interoception to conceptual representation of body interior
Source: Front Psychol. 2024 Jun 24;15:1389463. doi: 10.3389/fpsyg.2024.1389463 (PMC11229774; doi:10.3389/fpsyg.2024.1389463)
Supplement: Supplementary file 2 [file Data_Sheet_2.docx]

**Supplementary File**

**Inside-of-the-Body drawing task: Procedure and scoring**

**Procedure**

The task was adapted from the design of Tait & Ascher's Inside-of-the-Body test (1955).

Participants were asked to draw the inside of the body, including all organs and body parts, and then label the body parts drawn. The instructions were as follows:

*"Please draw the inside of the body, including all the organs and body parts. Next, please label the body parts you have drawn."*

Participants were informed that they had a maximum of 5 minutes to complete the drawing. The drawing was stopped after 5 minutes if the participant did not finish. The body parts drawn could be labeled after the 5-minute drawing period. Drawings were made with a ballpoint pen on a sheet of A4 paper presented vertically that contained the outline of a person (Appendix A1). After the drawing was labeled, the participant was asked "Can you describe what [name of body part] is used for or what function it is associated with?". The verbatim answers were recorded.

**Scoring**

Scoring included two evaluations: completeness/accuracy of the drawing and organization of the system.

***Scoring of completeness/accuracy of the drawing:***

Each organ/body part drawn and labeled was scored. If an item drawn was not correctly labeled, it should not be scored. For example, if the intestines are drawn and recognizable but labeled as the stomach, they should not be scored. Muscle- or bone-specific names were not expected.

Each organ/body part drawn was categorized into a physiological system (Table 1). Then, the drawings were scored according to the completeness/accuracy (slightly distorted, distorted/schematic, or blurred) and the localization (good or poor) of the body parts (Table 2).

- Completeness/accuracy level of the drawing

Slightly distorted:

- Sketch or shape correctly drawn
- Drawing more complex than a diagram or geometric shape, e.g., bones/veins/trachea/esophagus/intestines shown in 2 dimensions
- Presence of details, e.g., folds of the intestine/asymmetry of the heart
- Proportional
- Lungs not necessarily connected

Distorted/schematic:

- The reduction of the body part is shown in a familiar pattern (e.g., ♥ for the heart)
- Organs shown as geometric shapes
- Veins or nerves shown as curves
- Bones shown as lines
- Incorrect proportions or orientations

Blurred:

- Vague delineation of body parts
- Little or not recognizable mass or spot
- Ambiguous details
- Only 1 body part shown for double organs (e.g., kidneys)

Special cases:

- For organs/body parts with similar morphology in the same system, score only one element. For example, if several different bones are drawn (e.g., humerus, radius, pelvis, and clavicles) rate only one bone (specifically, the best drawn bone).
- If several different muscles are drawn (e.g., biceps, triceps, and abdominal muscles) rate only one muscle (specifically, the best drawn muscle).
- If veins and arteries and/or blood vessels are drawn, score only one element.
- For sensory organs, only score the interior ducts (e.g., nostrils, ear canal, tongue, eyeball, and eardrum). The nose, ears, and mouth are considered external elements and are not scored.

Localization

The evaluation of the location of organs was determined from a transparent outline of a person on which different drawing areas were delineated (Appendix A2).

- Good: correct position in the body using the scoring transparency (Appendix A2) (e.g., brain and tongue in the head, heart and lungs in the chest, stomach and liver in the belly; bones/muscles/veins/nerves can be represented in any location) or correct position in relation to the surrounding organs (e.g., heart near the lungs, stomach near the intestines). For double organs such as the lungs or kidneys, the organ was considered well located if one organ is found on each half (divided by a vertical axis) of the figure.
- Poor: body part drawn in the wrong place (e.g., bladder in the middle of the belly, heart in the belly, stomach in the chest) or 1/3 of the body part drawn was outside of the associated area.

**Table 1: Example of drawn body parts and associated physiological systems**

| **Organs/body parts drawn** | **Associated physiological system** |
| --- | --- |
| Brain, spinal cord, hypothalamus, cerebellum, nerves | Central nervous system (CNS) |
| Heart, arteries, veins, carotid artery, aorta, vena cava, coronary artery, ventricles, atria | Cardiovascular (C) system |
| Lungs, trachea, larynx, vocal cords | Respiratory (R) system |
| Teeth, esophagus, stomach, nasopharynx, pharynx, gallbladder, pancreas, appendix, large intestine/small intestine/intestines/digestive tract, colon, rectum, spleen, liver | Gastrointestinal (Gi) system |
| Bones, ribs, patella, joint, hip, rib cage, cartilage, jaw, muscles/thigh/gluteus, tendons, diaphragm | Musculoskeletal (MS) system |
| Kidneys, bladder, sphincter | Urinary (U) system |
| Uterus, ovaries, fallopian tubes, genital organs, vagina | Genital (G) system |
| Eye, eyeball, tongue, ear canal, nasal cavity, mouth, skin | Sensory (S) system |

**Table 2: Quantitative scoring of Inside-of-the-Body drawing**

| **Organ/body part** | **Physiological system** | **Slightly distorted** | | **Distorted/schematic** | | **Blurred** |
| --- | --- | --- | --- | --- | --- | --- |
|  |  | **Well located** | **Poorly located** | **Well located** | **Poorly located** |  |
|  |  | **2.5** | **2** | **1.5** | **1** | **0.5** |
|  |  |  |  |  |  |  |
| *Brain* | *CNS* |  | *x* |  |  |  |
| *Heart* | *C* |  |  | *x* |  |  |
| *Stomach* | *Gi* |  |  | *x* |  |  |
| *Liver* | *Gi* |  |  |  |  | *x* |
| *Intestines* | *Gi* | *x* |  |  |  |  |
| *Bones* | *MS* |  |  | *x* |  |  |
| *Kidneys* | *U* |  |  |  | *x* |  |
| *Uterus* | *G* |  |  | *x* |  |  |
|  | **Score** | ***2.5*** | ***2*** | ***6*** | ***1*** | ***0.5*** |
|  | **Total score of the drawing** | ***12*** | | | | |

*Scoring of the organization of systems:*

This score takes into account the number of organs/body parts drawn and the organization of the body parts. For each physiological system, the number of organs/body parts drawn was recorded, considering all distinct organs or body parts.

For example, a participant drew and labeled the "clavicle", "humerus", "radius", "bone" (labeled given to a leg bone) and "pelvis"; thus, we counted 5 elements of the musculoskeletal system. Only one bone was counted if he labeled two humerus bones, two radius bones, or two clavicle bones, etc.

He drew the "brain"; thus, 1 element was scored for central nervous system. He also drew the "liver", "stomach", "small intestine", "large intestine", leading to a score of 4 for the gastrointestinal system.

Notes for bones:

Count the spine as one bone, even if all the vertebrae are drawn.

Count the ribs as one bone.

Count the finger bones as only one bone if one bone is drawn in each finger; count them as two bones if two bones are drawn in each finger (Figure 1).

For each system in which at least 2 organs/body parts are represented, note the organization of these elements with each other (organized or scattered).

- Organized: body parts in the same system circuit are connected (e.g., trachea with the lung; esophagus with the stomach and intestines, ovaries with the uterus).

- Scattered: elements of the system are disconnected, scattered or distant.


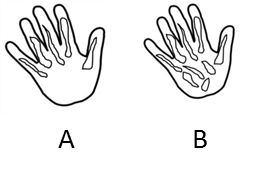


Fig. 1: Score of finger bones (A: scored as one bone; B: scored as two bones)
